# Improved Osteoblast and Chondrocyte Adhesion and Viability by Surface-Modified Ti6Al4V Alloy with Anodized TiO_2_ Nanotubes Using a Super-Oxidative Solution

**DOI:** 10.3390/ma8030867

**Published:** 2015-03-02

**Authors:** Ernesto Beltrán-Partida, Aldo Moreno-Ulloa, Benjamín Valdez-Salas, Cristina Velasquillo, Monica Carrillo, Alan Escamilla, Ernesto Valdez, Francisco Villarreal

**Affiliations:** 1 Facultad de Odontología Mexicali, Universidad Autónoma de Baja California, Av. Zotoluca y Chinampas, s/n, Mexicali C.P. 21040, Baja California, Mexico; E-Mail: beltrane@uabc.edu.mx; 2 Instituto de Ingeniería, Universidad Autónoma de Baja California, Blvd. B. Juárez y Calle de la Normal s/n, Mexicali C.P. 21280, Baja California, Mexico; E-Mails: monica@uabc.edu.mx (M.C.); alan.escamilla@uabc.edu.mx (A.E.); 3 School of Medicine, University of California San Diego, 9500 Gilman Drive, La Jolla, CA 92093, USA; E-Mails: aldiux_1@yahoo.com.mx (A.M.-U.); fvillarr@ucsd.edu (F.V.); 4 Instituto Nacional de Rehabilitación, Calz. México Xochimilco, No. 289, Arenal de Guadalupe, México C.P. 14389, D.F., Mexico; E-Mail: mvelasquillo@ciencias.unam.mx; 5 Sección de Estudios de Posgrado e Investigación, Escuela Superior de Medicina, Instituto Politécnico Nacional, Plan de San Luis y Díaz Mirón, México C.P. 11340, D.F., Mexico; 6 Centro Medico Ixchel, Bravo y Obregón, Mexicali C.P. 21000, Baja California, Mexico; E-Mail: ixchelcentromedico@hotmail.com

**Keywords:** titanium, TiO_2_ nanotubes, chondrocyte, osteoblasts, adhesion, tissue engineering

## Abstract

Titanium (Ti) and its alloys are amongst the most commonly-used biomaterials in orthopedic and dental applications. The Ti-aluminum-vanadium alloy (Ti6Al4V) is widely used as a biomaterial for these applications by virtue of its favorable properties, such as high tensile strength, good biocompatibility and excellent corrosion resistance. TiO_2_ nanotube (NTs) layers formed by anodization on Ti6Al4V alloy have been shown to improve osteoblast adhesion and function when compared to non-anodized material. In his study, NTs were grown on a Ti6Al4V alloy by anodic oxidation for 5 min using a super-oxidative aqueous solution, and their *in vitro* biocompatibility was investigated in pig periosteal osteoblasts and cartilage chondrocytes. Scanning electron microscopy (SEM), energy dispersion X-ray analysis (EDX) and atomic force microscopy (AFM) were used to characterize the materials. Cell morphology was analyzed by SEM and AFM. Cell viability was examined by fluorescence microscopy. Cell adhesion was evaluated by nuclei staining and cell number quantification by fluorescence microscopy. The average diameter of the NTs was 80 nm. The results demonstrate improved cell adhesion and viability at Day 1 and Day 3 of cell growth on the nanostructured material as compared to the non-anodized alloy. In conclusion, this study evidences the suitability of NTs grown on Ti6Al4V alloy using a super-oxidative water and a short anodization process to enhance the adhesion and viability of osteoblasts and chondrocytes. The results warrant further investigation for its use as medical implant materials.

## 1. Introduction

An imperative goal in bone and cartilage regeneration is the refinement of biomaterials with physicochemical and biological properties that better resemble the native architecture of human tissues to regenerate. Thus far, Ti and its alloys have frequently been used for dental and orthopedic implants because they offer good biocompatibility, low toxicity, corrosion resistance and favorable mechanical properties [1,2,3,4,5,6,7,8]. In this regard, the Ti6Al4V alloy offers superior physical and mechanical properties than commercially pure Ti (Cp-Ti), as well as excellent biocompatibility [9]. However, Ti- and its alloy-based implants occasionally loosen and fail [10]. Some of the factors that appear to lead to clinical failure are incomplete integration of the surrounding bone (*i.e*., osseointegration) and cartilage with the material implanted [11,12]. Interestingly, it has been suggested that by improving the osseointegration on the material, long-term implant failures will decrease [13]. The manipulation of cell-surface material interactions can have a profound effect in osseointegration. To enhance this process, several techniques focused on the biomaterial surface have been investigated, such as grit blasting, acid etching, anodic oxidation, molecule immobilization, sol-gel and calcium phosphate coating [3,14,15,16,17,18,19]. It is well known that anodic oxidation has been shown to provide a strong adherence of an NTs layer to the material surface [20,21], increasing material corrosion resistance [22,23,24], surface area [25,26,27] and, more importantly, bone growth [25] and cartilage adhesion [22].

Various studies suggest that nanostructured biomaterials may provide physical/chemical properties that resemble the native bone structure and, thus, may offer ideal substrates to support bone regeneration [28]. It is worth noting that the NTs’ diameter on the material surface has a significant effect on bone and cartilage integration [8,20,23]. For example, it has been reported that cultured preosteoblasts on NTs with a 70–100 nm diameter elicit good cell adhesion and selective differentiation into osteoblast-like cells when compared to smaller NTs (~30 nm) [29]. Moreover, NTs of 70–100 nm in diameter exhibit higher alkaline phosphatase activity levels, suggesting greater bone-forming ability with a superior filopodia elongation than smaller NTs [30]. Filopodia are highly dynamic structures containing actin filaments [31]. They play an important role in osteoblasts adhesion and act as a sensory organelle for cellular spreading and actin polymerization [32,33]. Nonetheless, previous reports suggest that the anodization process using fluoride as an electrolyte to obtain NTs of 80–100 nm diameter requires periods of 1 h [34,35] and perhaps 2 h [20]. According to this fact, we hypothesize that using a fluoride solution dissolved in super-oxidative water will accelerate the fabrication of NTs, which, in turn, will positively impact adhesion, proliferation and viability of primary osteoblasts and chondrocytes.

To our knowledge, this is the first study aimed at fabricating and characterizing NTs on the Ti6Al4V surface by anodic oxidation using a novel short process constituted by a commercial super-oxidative water used for medical instrument disinfection [36,37], containing ammonium fluoride, and studying its biological effects on primary pig periosteal osteoblast (PPO) and elastic cartilage pig chondrocytes (PCC). Cell morphology was examined by SEM and AFM. Cell adhesion and viability were evaluated by means of fluorescence microscopy.

## 2. Results

### 2.1. Surface Characterization

#### 2.1.1. SEM Material Characterization

**Figure 1:**
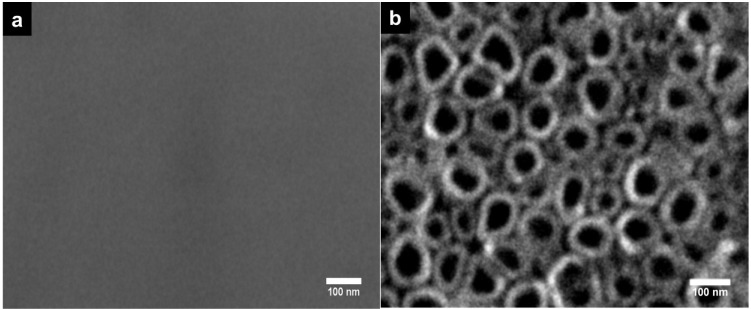
SEM micrographs of the experimental Ti6Al4V alloys. (**a**) Non-anodized flat Ti6Al4V alloy; (**b**) anodized Ti6Al4V alloy with NTs.

The non-anodized Ti6Al4V alloy surface features are displayed in Figure 1a, which shows, as expected, the smooth texture of the material. Following electrolytic anodization, a nanotubular and uniformly distributed layer was formed over the Ti6Al4V alloy surface, as noted by SEM examination (Figure 1b). The estimated diameter of the nanotubes was between 80 and 90 nm.

#### 2.1.2. Chemical Composition

Chemical composition analysis suggests that NTs formed on the anodized Ti6Al4V alloy due to the presence of increased oxygen content compared to the non-anodized alloy (Table 1). There is some contamination of fluoride in the anodized alloy due to the electrolytes used for anodization.

**Table 1:** Surface elemental compositions.

Sample	C (%)	N (%)	Al (%)	Ti (%)	O (%)	F (%)
Anodized Ti6Al4V	3.9	-	5.40	61.91	25.51	3.28
Non-anodized Ti6Al4V	3.45	3.38	6.06	87.11	-	-

#### 2.1.3. Surface Roughness

**Figure 2:**
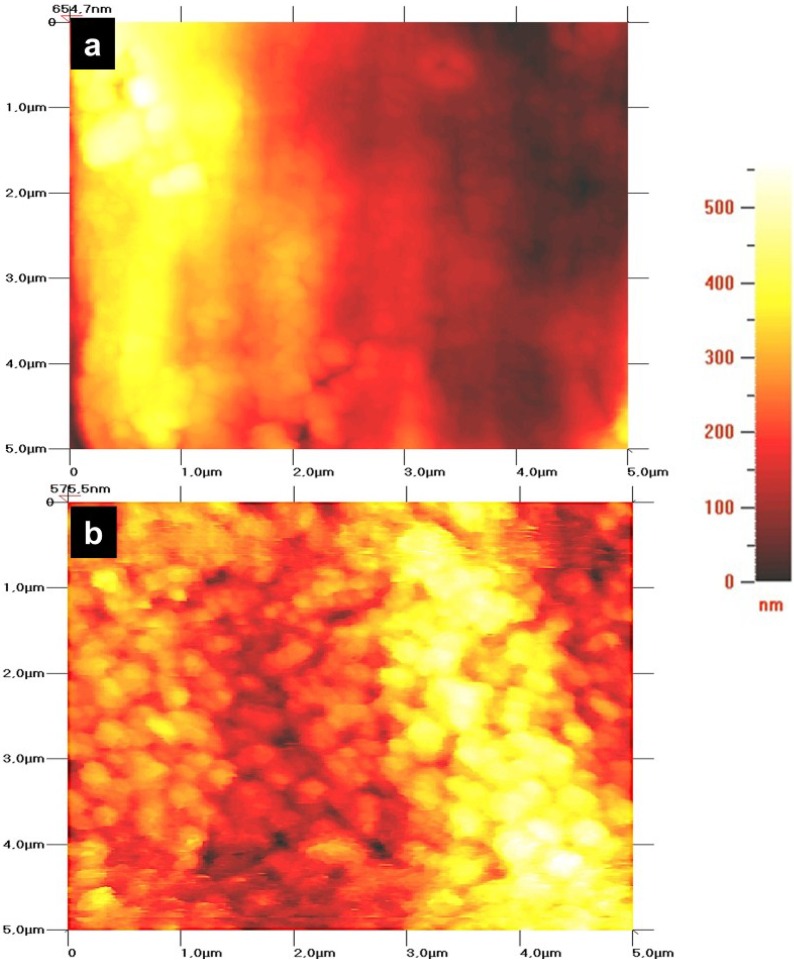
AFM micrographs of the Ti6Al4V alloy surface. (**a**) Non-anodized Ti6Al4V alloy, showing a smooth surface; (**b**) anodized Ti6Al4V alloy, showing a rougher surface. The scan area is 25 µm^2^. The color bar represents the surface height.

Representative AFM images of non-anodized and anodized Ti6Al4V alloys are shown in Figure 2. Non-anodized alloys show a flat and smooth surface compared to the anodize alloys (Figure 2a). Unfortunately, tube mouths could not be resolved, due to convolution with the probe tips used, which were not ultra-sharp; however, anodized alloy surfaces show increased roughness (Figure 2b), which is clearly associated with the tubular porous surface (represented by spots).

### 2.2. Biological Activity

#### 2.2.1. Biocompatibility Assay

**Figure 3:**
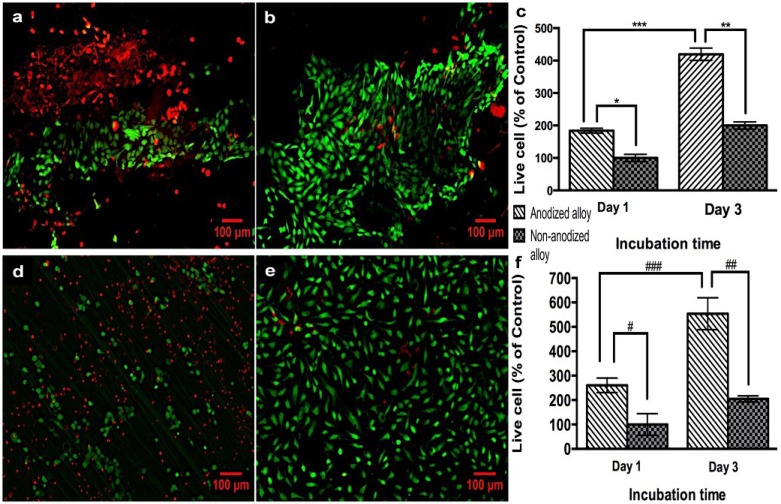
Live/dead staining of pig periosteal osteoblast (PPO) and pig cartilage chondrocytes (PCC) cells on the samples. (**a**) PCC cells on non-anodized Ti6Al4V alloy; (**b**) PCC cells on anodized Ti6Al4V alloy; (**c**) cell counting of live PCC cells; (**d**) PPO on the non-anodized Ti6Al4V alloy; (**e**) PPO on anodized Ti6Al4V alloy; (**f**) cell counting of live PPO cells. (Values are the mean ± SD, n = 3; * *p* < 0.05 and ^#^ *p* < 0.05, significantly different from non-anodized Ti6Al4V at Day 1 of cell growth; ** *p* < 0.05 and ^##^ *p* < 0.05, significantly different from non-anodized Ti6Al4V at Day 3 of cell growth; *** *p* < 0.05 and ^###^ *p* < 0.05, significantly different from anodized Ti6Al4V at Day 3. All the images are the same magnification. The bar is 100 μm).

PPO and PCC cellular viability was evaluated by using a live/dead staining kit (Figure 3). Representative fluorescence images of PCC cells grown on non-anodized and anodized alloy sheets for three days are shown in Figure 3a,b, respectively. After one and three days of PCC cell growth on anodized alloy, an increased percentage of live cells was observed when compared to the non-anodized alloy (Figure 3b,c). Moreover, in the non-anodized alloy, after three days of PCC cell growth, a lower number of live cells compared to the anodized alloy was observed, suggesting cell toxicity (Figure 3c). Significant differences were observed at Day 1 and 3 in the anodized alloy, suggesting greater PCC proliferation. Representative images of PPO cells after three days of growth on non-anodized and anodized alloys are shown in Figure 3d,e, respectively. The quantification of live PPO cells (Figure 3f) indicates a higher number of live PPO cells on the anodized alloy at both days as compared to the non-anodized material. Moreover, differences were observed in the live PPO cell percentage within the anodized alloy groups at Day 1 and Day 3, as observed for PCC analysis. In addition, the dead PPO cell number was increased on the non-anodized alloy compared to the anodized material, as observed in Figure 3d.

#### 2.2.2. Cell Adhesion

**Figure 4:**
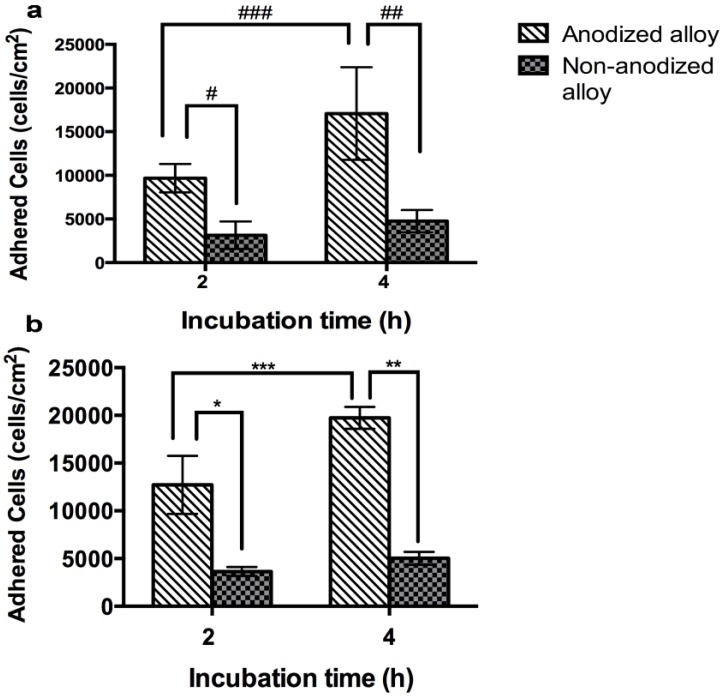
Cell counting using nuclei (DAPI) staining for PPO and PCC cells. (**a**) PPO and (**b**) PCC adhesion. (Values are the mean ± SD, n = 3; ^#^ *p* < 0.05 and * *p* < 0.05, significantly different from non-anodized Ti6Al4V alloy at 2 h of cell growth; ^##^ *p* < 0.05 and ** *p* < 0.05, significantly different from non-anodized Ti6Al4V at 4 h of cell growth; ^###^ *p* < 0.05 and *** *p* < 0.05, significantly different from anodized Ti6Al4V at 4 h).

The cell adhesion results of PPO grown on non-anodized and anodized Ti6Al4V alloys are illustrated in Figure 4a. The data show increased PPO cell number on the anodized alloy compared to the non-anodized material at both days of culture. Figure 4b indicates the PCC cell number at 2 h and 4 h of cell growth on the non-anodized and anodized alloys. The data show an increased cell adhesion on the anodized material at both times of culture when compared to the non-anodized alloy.

#### 2.2.3. Cell Morphology by SEM

**Figure 5:**
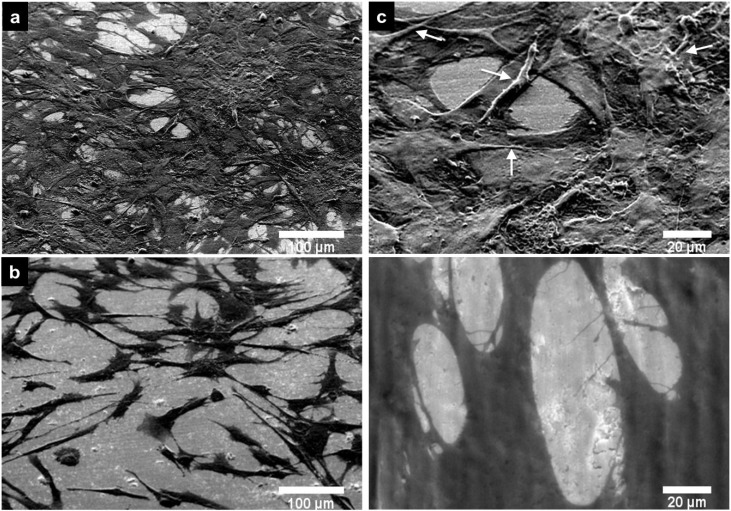
SEM micrographs of PPO cells at Day 3 of cell growth on the Ti6Al4V alloys. (**a**) PPO on anodized Ti6Al4V alloy; (**b**) PPO on non-anodized Ti6Al4V alloy; (**c**) PPO on anodized Ti6Al4V alloy (high magnification); (**d**) PPO on the non-anodized Ti6Al4V alloy (high magnification). White arrows denote filopodia.

**Figure 6:**
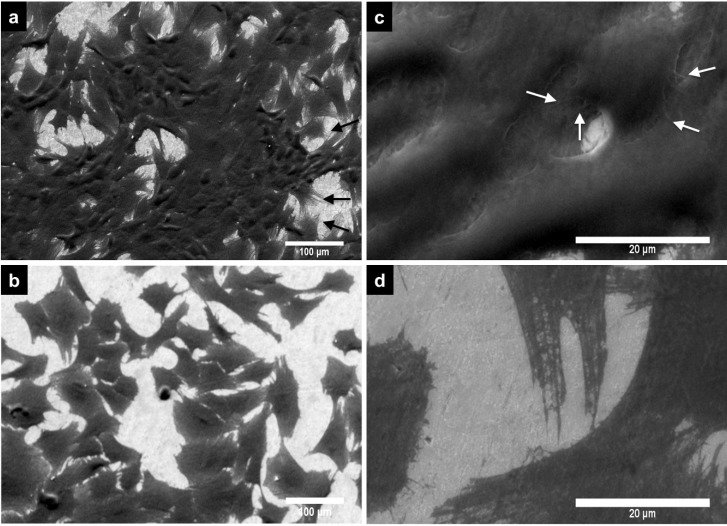
SEM images of PCC cell at Day 3 of cell growth on the Ti6Al4V alloys. (**a**) PCC on anodized Ti6Al4V alloy; (**b**) PCC on the non-anodized Ti6Al4V alloy; (**c**) PCC on anodized Ti6Al4V alloy (high magnification); (**d**) PCC on the non-anodized Ti6Al4V alloy (high magnification). White arrows denote filopodia.

Figure 5 shows images of PPO cells at three days grown on non-anodized and anodized Ti6Al4V alloys. On the non-anodized alloy, PPO cells show a flatter appearance, elongated spread (which denotes a fibroblast like shape) (Figure 5b) and lower intercellular connections compared to cells on the nanostructured Ti6Al4V material (Figure 5a). Moreover, the anodized material enhanced the osteoblast-like morphology, which indicates a greater number of cellular interconnections, and anchored filopodia to the material surface (Figure 5c). Additionally, the filopodia morphology observed on the anodized alloy had a very dense fibril-type form, which was lacking in cells grown on the non-anodized alloy (Figure 5d). The PCC morphology analyzed after three days in culture on the alloys is presented in Figure 6. On the anodized material, PCC cells (Figure 6a) present a greater number of intercellular connections and an elongated filopodia formation compared to the non-anodized material (Figure 6b). Moreover, at higher magnification, cell bodies can be observed with an increased number of cellular filopodia anchored to the nanostructured material surface (Figure 6c) *vs.* the translucent and thinner filopodia present on the non-anodized material (Figure 6d). Interestingly, on the non-anodized and anodized alloy, a similar cellular polygonal shape is observed, although the cell density is higher on the nanostructured surface material.

#### 2.2.4. Cell Morphology by AFM

**Figure 7:**
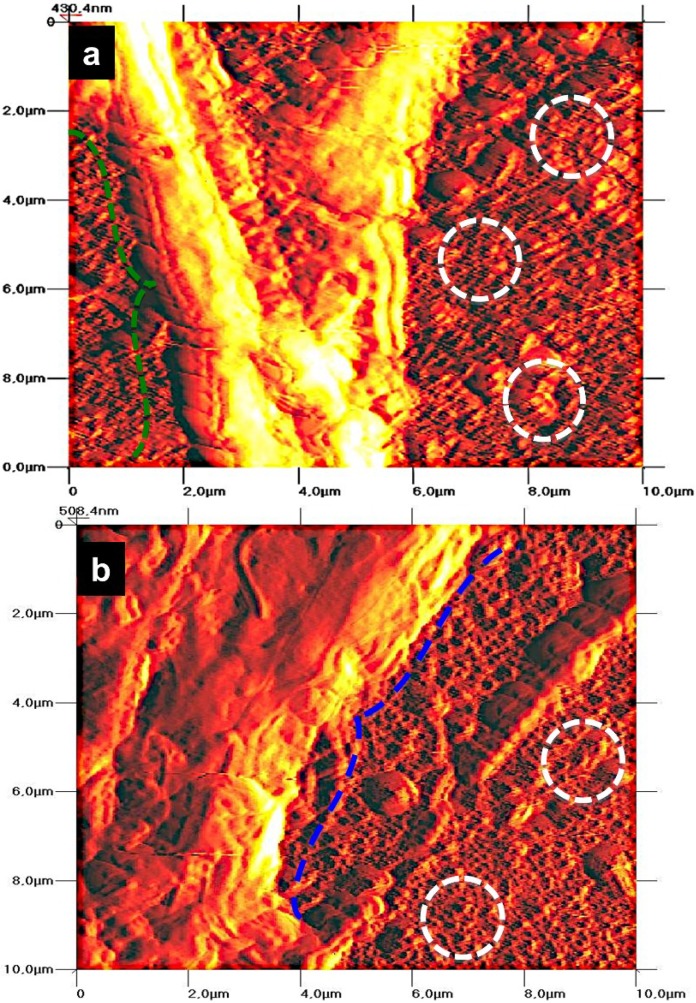
AFM micrographs for PPO and PCC cells at Day 3 of cell growth on anodized Ti6Al4V alloy. (**a**) PPO; (**b**) PCC. (The green dashed arrow indicates cellular filopodia; the blue dashed arrow indicates monolayer-like morphology; circles represent the NTs’ surface).

The PPO and PCC cell morphology after three days in culture on the anodized alloys are displayed in Figure 7. PPO cells cultured on the nanostructured material show the evident formation of filopodia (green dotted arrow) anchored to the material surface (Figure 7a), which indicates an evident morphological modification that denotes a fibril-type form (associated with cellular filopodia) compared to the uniform tubular porous (white dotted circles). Figure 7b shows morphological alterations, suggesting a monolayer-like morphology (blue dotted arrow) around the material surface.

## 3. Discussion

Enhancing tissue development around grafts is a process that involves adhesion, proliferation, secretion of extracellular matrix (ECM) proteins and mineralization for bone formation [5]. It is well known that the interaction between cells in our body and the ECM is necessary for optimal cell function and support [25]. Interestingly, this communication between the cells and the ECM is suggested to potentially occur at a nanoscale level. Indeed, nanostructured surface materials have been suggested to impact cell physiology in the same manner as the natural ECM [38]. Specifically, various studies have reported that nanostructured surface modifications on Ti and its alloys exhibit enhanced effects on cell adhesion, proliferation and bone formation, which, as a consequence, results in good osseointegration [39,40].

Thus far, Ti has been the most studied biomaterial with surface modifications, specifically NTs’ effects on osteoblast cell biology *in vitro*. However, the effects of NTs on Ti6Al4V alloy built by anodization using a commercial super-oxidative water with fluoride have not been investigated. Using this novel electrolyte allows us to obtain 80 nm NTs with just only 5 min of anodization (Figure 1b) with similar morphology as reported by Wang *et al.*, who anodized for 1 h [34], and Narayanan *et al.* for 2 h [20] with fluoride and deionized water as the electrolyte. Moreover, Yao *et al.* reported that using 0.5% HF and 20 V, a nanotube-like structure appeared between 2 and 5 min of anodization and was completely observed at 20 min [35], while here, we observed the NT’s morphology in 5 min, probably due to the presence of the oxidative components that, with fluoride, synergistically act, promoting a faster oxidation, which leads to a nanostructured morphology.

In a recent study, Stan *et al.* suggested that NTs on Ti6Al4V alloy enhanced G292 osteoblast cell growth when compared to anodized Ti6Al7Nb alloy [41]. Likewise, in another study, Saharudin *et al.* suggested that NTs on Ti6Al4V alloy increased PA6 cell adhesion and viability when compared to the non-anodized material [42]. Therefore, considering the aforementioned enhanced cellular response on nanostructured Ti6Al4V alloy and the improved corrosion resistance, as well as wear resistance, we investigated the biological effects that our NTs grown on Ti6Al4V alloy have on cell adhesion and viability.

In agreement with the aforementioned studies on nanostructured Ti6Al4V alloy, we have evidenced that our NTs enhance PPO cell adhesion. Importantly, osteoblast cell adhesion on the material surface is necessary for promote bone building [38]. However, adhered cells on the material surface do not suffice as a good indication of proper osseointegration; also, viable osteoblasts are necessary to sustain the functionality during regeneration and eventually integrate into the host tissue [43]. Indeed, our results also suggest increased osteoblast cell viability at one day and three days of culture on the anodized alloy compared to the non-anodized material, suggesting a higher number of osteoblasts capable of interacting and perhaps integrating in the long term with the material. A possible explanation for the increased osteoblast adhesion and viability is that NTs increase the surface area and decreased the wettability [2,30,44], a property widely observed for NTs obtained by anodization, which, in turn, may promote an elevated attachment of ions, small molecules and proteins, enhancing a higher cell-cell interaction via ECM proteins and, thereby, anchoring a major number of cellular filopodia to the nanostructured surface [29,45,46,47]. Filopodia act as a partial regulator of cell adhesion, proliferation and cell-cell interactions [48] and have been associated with increased alkaline phosphatase activity and a superior rate of Ca and P secreted by osteoblasts cultured on NTs, as reported by others [30,49]. This notion is supported based on our findings that show a greater number of cellular interconnections and a higher number of anchored cellular filopodia on the NTs (Figure 5a,c); whereas, the osteoblasts grown on the non-anodized material have poor cellular interconnections, flat and spread elongation, which denotes a fibroblast-like shape, decreased dissemination, and lack filopodia propagation (Figure 5b,d), suggesting concordance with previous reports [30,38]. Besides, it is well documented that NTs have increased surface roughness, a property observed in this study (Figure 2), which elicits increased osteoblast adhesion, proliferation and a well-supported osteoblast-like morphology, as reported in various studies [30,44,46].

During endochondral bone formation, chondrocytes promote mineralization, which, in turn, serves as a template for bone deposition [50]. On the other hand, the inability of chondrocytes to adhere and consequently create new cartilage tissue on the biomaterial used for regenerative purpose has remained a considerable problem [51]. Despite the important role that chondrocytes have in bone and cartilage regeneration, only a few reports exist addressing this point in nanomaterial research. A study by Burns *et al.* reported the effects that nanotubular-anodized Ti has on human articular chondrocytes, whereby he suggested increased cell adhesion at 4 h of cell culture on NTs [22]. Moreover, Brammer *et al.* described enhanced glycosaminoglycan secretion, as well as increased collagen II transcription levels for chondrocytes incubated on anodized Ti [23]. Here, we evidence not only increased chondrocyte cell adhesion, but also cell viability at Day 1 and Day 3 of cell growth on anodized Ti6Al4V alloy. Furthermore, a superior number of intercellular connections and filopodia are observed in chondrocytes cultured on NTs (Figure 6a,b), while on non-anodized alloy, we found a flat cellular morphology and decreased number of filopodia anchored to the material surface (Figure 6b,d), as suggested in earlier studies [23,52].

It is worth noting that the dimension of anodized nanotubes varies between the scientific literature and, so, the biological effects on cells. Brammer *et al.* suggested that NTs with a diameter between 70 and 100 nm evoke increased elongated MC3T3-E1 (mouse osteoblast) cellular morphology, nuclei and alkaline phosphatase activity, suggesting greater bone-forming capability when compared to NTs with smaller diameters [30]. Li *et al.* evidenced that 70 nm diameter NTs grown on Ti by anodic oxidation enhanced MC3T3-E1 cell adhesion and proliferation at four and seven days of culture compared to non-anodized and nanopore-based Ti [27]. Moreover, Smith *et al.* provided evidence that NTs grown on Ti with an average diameter of 70–90 nm showed the highest human dermal fibroblasts and human epidermal keratinocyte adhesion and viability at four days in culture when compared to non-anodized Ti [53]. Our results provide further evidence that our 80 nm diameter NTs grown on Ti6Al4V alloy have considerable beneficial effects on osteoblast cell adhesion and viability, when compared to non-anodized alloy. Furthermore, our data suggest that our nanostructured surface increases the chondrocyte cell adhesion, proliferation and viability. Overall, this study provides evidence of anodic oxidation using super-oxidative water as a means to grow NTs on Ti6Al4V alloy and its increased biocompatibility on chondrocyte and osteoblast cells. Nonetheless, more research is needed in order to extrapolate these findings to *in vivo* experiments as a means to develop new materials capable of supporting bone and cartilage growth.

## 4. Experimental Section

### 4.1. Synthesis of NTs

Discs of Ti6Al4V (ASTM F-136; Supra Alloys Inc., Camarillo, CA, USA) with a 1.5 cm^2^ surface area were polished using SiC emery paper (100 to 2000 grit) and 1 micron alumina for finishing. Mirror finish disc surfaces were mounted in a special flat 125 mL cell and electrolytically anodized using Microdacyn 60 super-oxidative water (Oculus Technologies, Guadalajara, JAL, Mexico) at pH 6.8, containing 35.7 mg/L sodium hypochlorite (NaOCl), 25.2 mg/L hypochlorous acid (HClO), 100 mg/L sodium chloride (NaCl) and 10 mg/L ammonium fluoride (NH_4_F Sigma Aldrich). A 20 V potential was applied using a DC power supply for 5 minutes and a platinum mesh as the counter electrode. The process was carried out at room temperature. Finally, the discs were cleaned in an ultrasonic bath with distilled water for 5 minutes to eliminate residues of fluoride salts [35], rinsed with isopropyl alcohol and dried in a desiccator for 12 h. Non-anodized Ti6Al4V alloy discs were used as a control.

### 4.2. Substrate Surface Characterization

#### 4.2.1. SEM

The structural morphology of anodized and non-anodized Ti6Al4V alloy were examined by SEM (JSM-6010LA, JEOL, Peabody, MD, USA); the images were taken at a 15 kV accelerating voltage.

#### 4.2.2. EDX

Surface chemical compositions of anodized and non-anodized Ti6Al4V alloy were assessed using EDX (JSM-6010LA, JEOL) with a Silicon Drift Detector, connected to the SEM.

#### 4.2.3. AFM

AFM (Quesant Q-Scope 350, AMBIOS, Agura Hills, CA, USA) was used to evaluate the surface roughness of the anodized and non-anodized Ti6Al4V alloy. The process was performed at room temperature and ambient conditions; using an anti-acoustic box to prevent noises, which can affect measurements. Topographic images were obtained by operating the instrument in the contact mode. A 40-μm X-Y and 4-μm Z scanner equipped with silicon tips and 10 nm tip curvature were used. The experiment scan area was 25 μm^2^.

### 4.3. Biological Activity

#### 4.3.1. Cell Culture

PPO cells were obtained from pig femur periosteal bone. PCC cells were isolated from pig cartilage ear. The cells were cultured in DMEM-F12 (1:1) (Dulbecco’s Modified Eagle’s Medium-Ham’s F-12, Gibco-Invitrogen, Carlsbad, CA, USA), supplemented with 10% FBS (fetal bovine serum, Gibco-Invitrogen) and 1% penicillin/streptomycin (Gibco-Invitrogen) at 37 °C in 5% CO_2_. The culture media were changed every 3 days. The cells were seeded on the anodized and non-anodized Ti6Al4V alloys at a cell density of 25,000 cells/cm^2^, previously collocated in 12-well plates. Cells were grown for 1 and 3 days for subsequent biological analysis. To confirm chondrocyte phenotype, immunofluorescence of collagen type II was performed (data not shown). All experiments were conducted with cells at 1st–3rd passage to avoid loss of phenotype.

#### 4.3.2. Cell Viability

Cell viability was evaluated after 3 days of PPO and PCC cell growth using a live/dead viability/cytotoxicity assay kit (Molecular Probes; Gibco-Invitrogen, Carlsbad, CA, USA). This method is based on the determination of living and dead cells by two analyses: calcein-AM for esterase activity and ethidiumhomodimer-1 for plasma membrane integrity [54,55]. In brief, cells were incubated with a mixture of 1 mM calcein-AM and 2 mg/mL ethidium homodimer-1 for 45 min at 37 °C. Thereafter, specimens were inverted onto glass slides with fluorescence mounting medium (DAKO, Agilent Technologies, Carpinteria, CA, USA), examined and photographed using a green (live) and red (dead) filter under a fluorescence microscope (Axio Observer A1, Carl Zeiss, Thornwood, NY, USA). At least five fields of view were imaged at random, and cells were analyzed with the AxionVision software.

#### 4.3.3. Cell Adhesion

PPO and PCC cell adhesion was evaluated by fluorescence microscopy after 2 and 4 h of cell growth. At this time, non-adhered cells were removed by rinsing with phosphate buffered saline (PBS). Thereafter, cells were stained with 4’,6’-diamidino-2-phenylindole (DAPI; Molecular Probes, Carlsbad, CA, USA) for 5 min at room temperature and then washed with PBS. Finally, samples were inverted onto cover slips, mounted, visualized and photographed using a blue filter by a fluorescence microscope (Axio Observer A1, Carl Zeiss). Cell number was obtained from five random fields using a fluorescence microscope (Axio Observer A1, Carl Zeiss).

#### 4.3.4. Cell Morphology by SEM

After 3 days, cells on the anodized and non-anodized Ti6Al4V alloy were fixed with 5% (w/v) glutaraldehyde (Sigma, St. Louis, MO, USA) for 2 h at 25 °C. After fixation, they were washed three times with PBS (10 min) each wash. Then, cells were dehydrated in grade series of alcohol (50%, 70%, 90% and 100%) for 30 min at each concentration. Finally, samples were sputter-coated with gold (50 nm gold layer) for 8 s. The morphology of PCC and PPO cells was observed under SEM.

#### 4.3.5. Cell Morphology by AFM

Following 3 days of PPO and PCC cell growth on the anodized alloy, AFM was also used to analyze cell morphology because of its high-resolution probe with an acceptable resolution in the sub-nanometer range [56]. The cells were examined by visual scanning at 1 Hz over a 100 μm^2^ region at a scale angle of 0°. All experiments were conducted at room temperature and ambient conditions over a 20 to 30 min period.

### 4.4. Statistical Analysis

Three independent experiments were performed, each in triplicate. Numerical data were analyzed using GraphPad Prism 6 (GraphPad Software Inc., La Jolla, CA, USA). The significance of differences between group means was determined using two-tailed unpaired Student’s *t-*test or one-way ANOVA followed by Tukey’s multiple comparisons test when appropriate. A *p* < 0.05 was considered statistically significant.
